# PhenoDEL: A Novel Screening Strategy Based on Intracellular Protein Degradation Activity

**DOI:** 10.1021/acschembio.6c00234

**Published:** 2026-08-13

**Authors:** Yuichi Onda, Yurika Ochi, Toshihiro Araki, Miho Kageoka-Takahashi, Shuzo Takeda, Kazunori Yamada, Takehiko Ueda, Ken Ohno, Minoru Tanaka, Daiki Sakai, Miki Hasegawa, Yoshihito Tanaka

**Affiliations:** † Tanabe Pharma Corporation, Kanagawa 227-0033, Japan; ‡ Nikon Corporation, Tokyo 140-8601, Japan

## Abstract

Targeted protein degradation (TPD), including proteolysis targeting chimeras (PROTACs) and molecular glue degraders (MGDs), is a promising therapeutic approach. However, systematic discovery of such small molecules remains a major challenge. Here, we present PhenoDEL, a novel phenotypic DNA-encoded library (DEL) screening platform that integrates one-bead one-compound DEL (OBOC-DEL) with the Beacon optofluidic system for single-cell analysis. By coculturing individual OBOC-DEL beads and engineered reporter cells in nanoliter-scale chambers, PhenoDEL enables time-resolved, single-cell phenotypic evaluation and direct linkage between compound identity and intracellular response. As a proof-of-concept, we demonstrate discrimination of active and inactive on-bead compounds using an FKBP12^F36V^-EGFP degradation reporter in PC-3 cells, followed by DNA barcode decoding.

## Graphical abstract

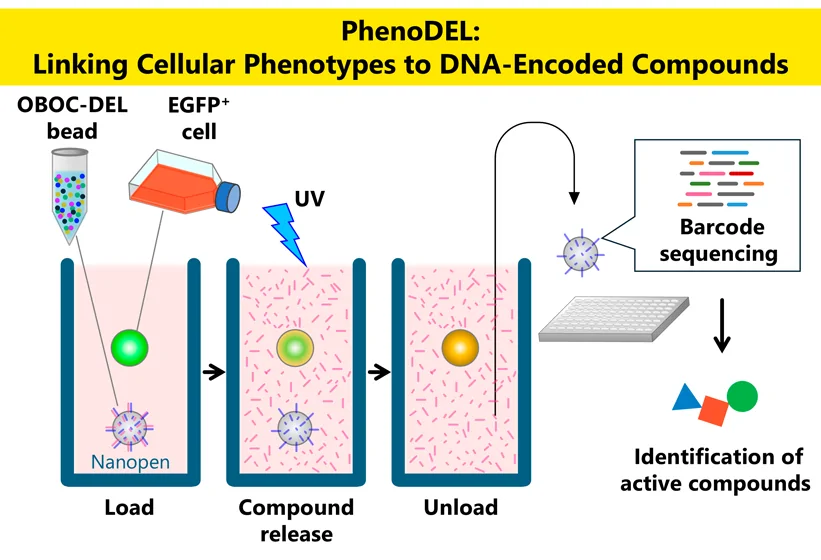

## Introduction

Targeted protein degradation (TPD) is emerging as a promising approach in new drug discovery and development, utilizing technologies like proteolysis targeting chimeras (PROTACs) and molecular glue degraders (MGDs).^1–3^ The systematic discovery of small molecules that induce proximity between proteins enables the targeted modulation and degradation of previously undruggable proteins, thereby expanding the scope of drug discovery to include a wide range of biological processes and molecular targets through event-driven pharmacology.^4,5^ PROTACs are heterobifunctional molecules that recruit E3 ligases (E3L) to degrade protein of interest (POI), while MGDs facilitate protein–protein interactions between POI and E3L to induce degradation. Although most MGDs have historically been discovered serendipitously through phenotypic or viability-based screens, recent advances in mechanistic understanding and assay technologies are enabling a shift toward more systematic and rational approaches for their identification.^6–9^ Accordingly, rational design and high-throughput screening (HTS) are becoming essential to improve the efficiency and specificity of MGD discovery, ultimately enhancing their therapeutic potential.

DNA encoded library (DEL) technology can evaluate a vast number of compounds (millions to billions) at once, enabling the discovery of ligands for undruggable targets that are difficult to target using conventional drug discovery methods such as HTS.^10–14^ DEL has been used not only for the discovery of binders, but also for the optimization of compounds, especially PROTACs that induce protein degradation, as well as for the discovery of MGDs.^15–18^ Beyond in vitro selections using purified proteins, cell-based DEL screening has also been reported. Li et al. demonstrated the DEL screening in a living cell using oocytes from the South African clawed frog *Xenopus laevis*.^19^ Recently, solid-phase DEL which is called one-bead one-compound DEL (OBOC-DEL) has been developed to further expand DEL screening from affinity screening to activity screening.^20–23^ (Figure 1A). Paegel and co-workers have reported an integrated droplet-based microfluidic circuit that directly screens OBOC-DEL (67,100 compounds) against phosphodiesterase autotaxin (ATX) for enzyme inhibitors.^22^ Paegel and Paulick have reported in vitro transcription–translation (IVTT) activity assay with GFP reporters fused to target genes, screening for inhibitors using OBOC-DEL (5348 compounds) in microfluidic droplets and the hits inhibited PCSK9-GFP IVTT and reduced PCSK9 levels in HepG2 cells.^24^ Scientists at Plexium and Amgen have described Picowell RNA-seq screening to identify The Von Hippel-Lindau (VHL) MGDs from a diverse VHL-focused OBOC-DEL (4327 compounds), discovering dGEM3, which targets GEMIN3 for degradation, highlighting the potential of VHL for novel target identification.^25^ Related patent disclosures describe OBOC-DEL formats and droplet- or microfluidics-enabled cellular screening platforms, which broaden the scope of cellular screening strategies.^26–29^

**Figure 1:**
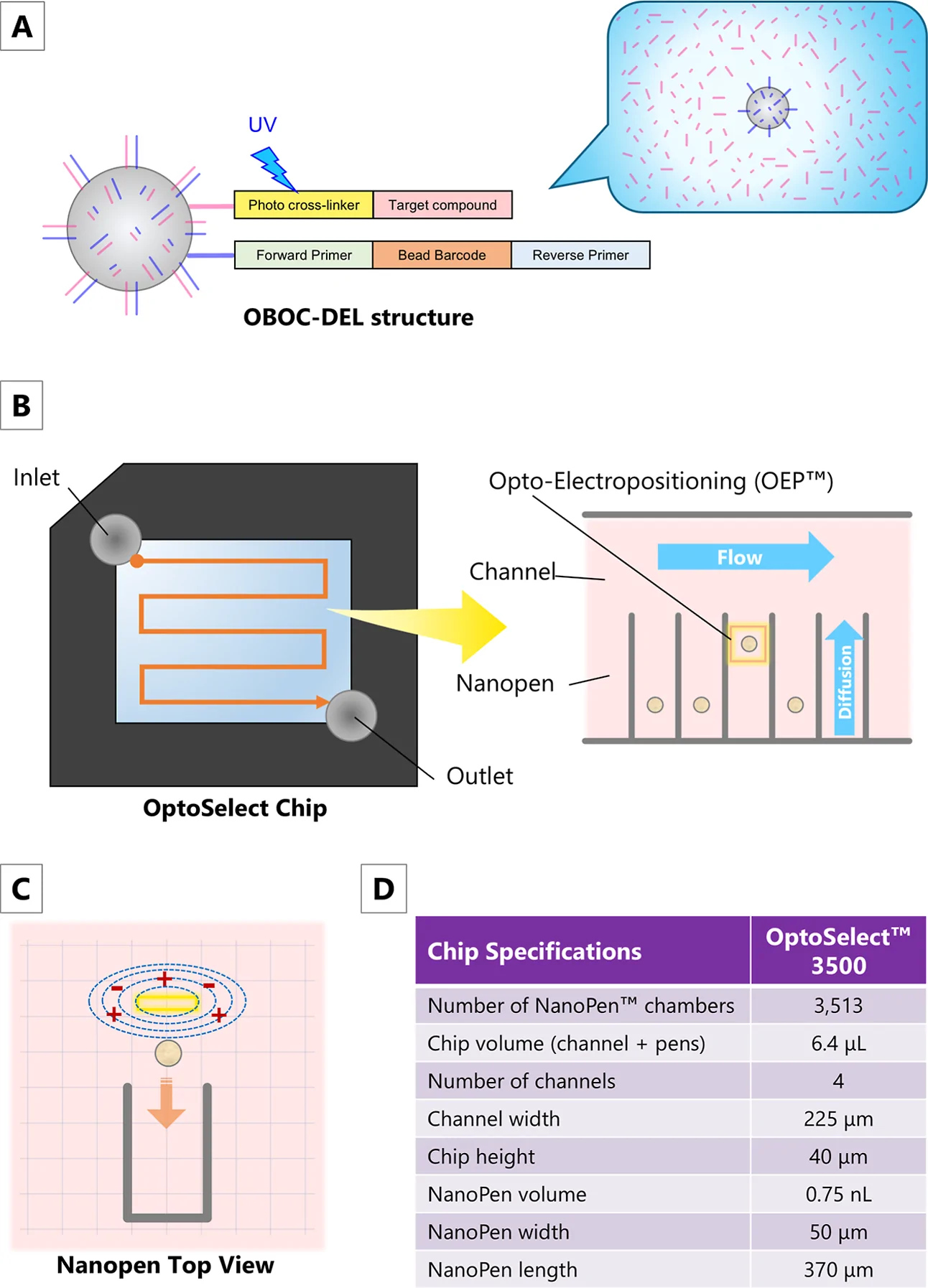
(A) One-bead one-compound DEL (OBOC-DEL) structure. The linker is cleaved by irradiation with near-ultraviolet light (390 nm) and the compound is released into the solution. (B) The OptoSelect chip used in the Beacon Optofluidic System (#110–08004/Bruker Corporation) has a structure called a nanopen chamber, which has a volume approximately 100,000 times smaller than a microwell, and a flow path called a channel, which are arranged on a semiconductor substrate. (C) Particles in the liquid loaded into the flow channel are “pushed” into the nanopen by a light-induced electric field using Opto-Electropositioning (OEP). Liquid exchange within the nanopen is achieved by diffusion. OEP optical technology nondestructively irradiates the OptoSelect Chip substrate with visible light to create a localized electric field gradient. Cells and particles in liquid are usually repelled by this electric field gradient. The Beacon system can move cells and particles within the chip by controlling the light pattern and electric field gradient. (D) The OptoSelect 3500 Chip (#750–00012/Bruker Corporation) used in our experiment has approximately 3500 nanopens with a volume of 0.75 nL. Up to four chips can be run simultaneously, theoretically allowing screening of more than 10,000 samples at a time.

The Beacon optofluidic system (currently distributed by Bruker Cellular Analysis) integrates optics, fluidics, and imaging to enable high-throughput single-cell manipulation and analysis.^30,31^ Its OptoSelect chip contains 3500 nanopen chambers (∼0.75 nL each), ∼ 100,000 times smaller than microwells, allowing screening of over 10,000 samples (Figure 1B, D). Cells are positioned using Opto-Electropositioning (OEP), a light-induced electric field for precise, noninvasive control (Figure 1C).

Initially developed for cell line development, Beacon achieves >99% single-cell origin verification, outperforming limiting dilution and fluorescence-activated cell sorting.^32^ In antibody discovery, it enables direct screening of antibody-secreting cells via bead-based fluorescence assays, identifying high-affinity clones without hybridoma generation.^33^ It also supports multiplexed functional assays for CAR-T and TCR-T cells, linking phenotype to genotype at single-cell resolution.^31,34^ While Beacon excels in screening large biomolecules like antibodies, applying it to small molecules requires additional engineering. Specifically, compounds must be individually delivered and retained in nanopen chambers to ensure localized exposure. To address this, we developed PhenoDEL—a phenotypic DNA-encoded library screening method that leverages Beacon’s single-cell resolution and compartmentalized architecture to identify small molecule degraders.

In this study, we introduce a novel screening concept termed PhenoDEL which combines OBOC-DEL technology with single-cell phenotypic analysis on the Beacon platform. Unlike conventional DEL approaches optimized for throughput and affinity-based enrichment, PhenoDEL adopts a conceptually distinct strategy that enables single-cell-resolved phenotypic screening. As a proof-of-concept, we applied PhenoDEL to screen for compounds that induce degradation of FKBP12^F36V^-EGFP fusion proteins in engineered PC-3 cells. This implementation enables direct observation and comparison of cellular phenotypes before and after compound release at the single-cell level—capabilities that are inaccessible to population-averaged assays. By excluding cells with low initial reporter activity and tracking dynamic phenotypic changes, this study demonstrates the feasibility and practicality of linking individual on-bead compounds to intracellular phenotypic responses using OBOC-DEL.

## Results and Discussion

To evaluate the PhenoDEL workflow, we employed a well-characterized FKBP12^F36V^-based degradation model. Use of a single, defined system enabled validation of the screening concept and clear attribution of observed phenotypes to the workflow.

### Concept of Single Bead Screening in Beacon

To enable phenotypic screening at single-cell resolution, we developed a workflow that integrates OBOC-DEL beads with FKBP12^F36V^-EGFP reporter cells using the Beacon Optofluidic System. In this workflow, each nanopen chamber on the OptoSelect chip was loaded with a single OBOC-DEL bead and a single PC-3 cell genetically engineered to express FKBP12^F36V^-EGFP fusion proteins (Figure 2). After loading, residual liquid in the channel was removed by CO_2_ flow, and compounds were then released from the beads by near-ultraviolet (UV-A) irradiation through a DAPI filter (excitation wavelength: 390/40 nm). The released compound diffused into the nanopen and interacted with the cocultured cell. Changes in EGFP fluorescence were monitored to evaluate compound-induced phenotypic effects. Beads associated with cells exhibiting EGFP fluorescence loss were collected and subjected to next-generation sequencing (NGS) to identify the compound via its DNA barcode. This single-bead, single-cell coculture strategy enabled a direct link between compound identity and cellular phenotype, providing a powerful platform for activity-based screening of DNA-encoded libraries.

**Figure 2:**
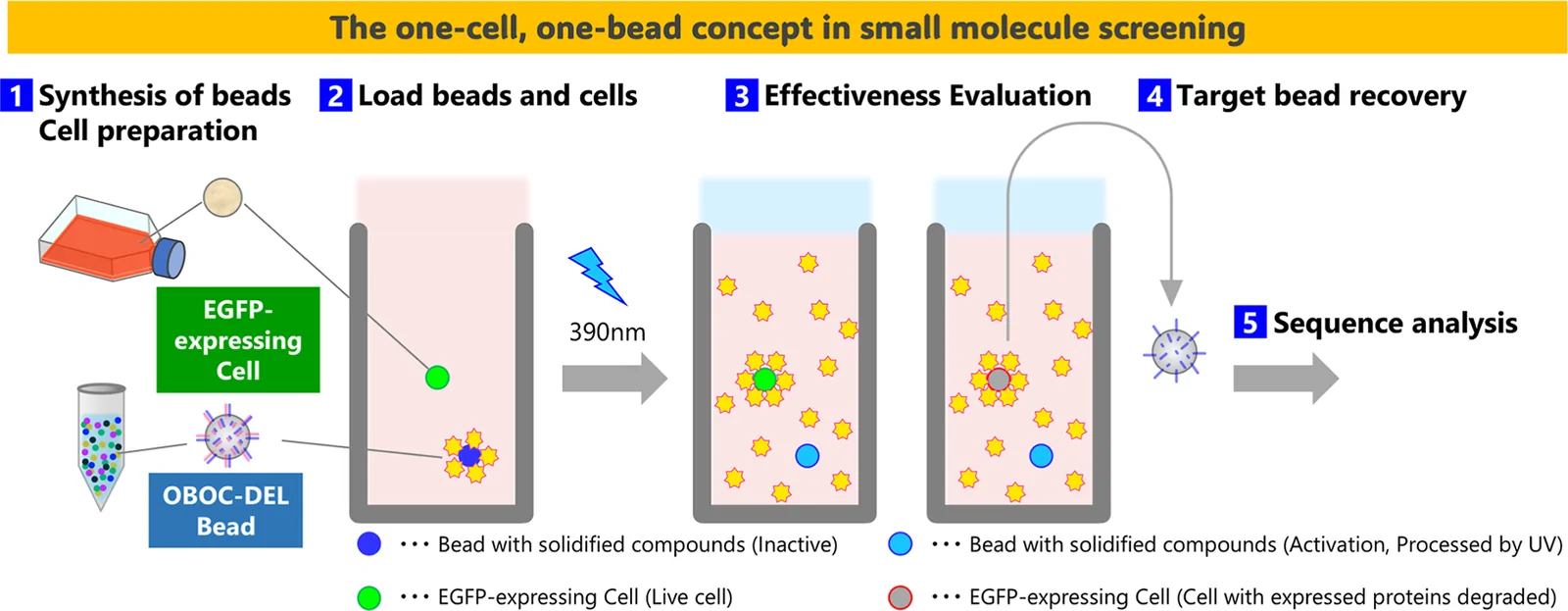
Concept of single bead screening on the Beacon platform: (1) OBOC-DEL beads synthesized for this experiment were suspended at a concentration of 1.5 × 10^6^ particles/mL in PBST. PC-3 cells engineered to express FKBP12^F36V^-EGFP fusion proteins were cultured for at least two passages to ensure acclimation and adjusted to 2.0 × 10^5^ cells/mL immediately prior to loading into the Beacon system. (2) A single bead was loaded into each nanopen, followed by the introduction of a single FKBP12^F36V^-EGFP-expressing PC-3 cell into the same chamber. After successful penning of both the bead and the cell, residual liquid in the channels was removed by CO_2_ flow for a defined period. (3) Near-ultraviolet (UV-A) light was irradiated through a DAPI filter (excitation wavelength: 390/40 nm) for 200 ms to cleave the compound from the bead. The released compound diffused into the nanopen and interacted with the cocultured cell. Time-lapse imaging was performed to monitor changes in EGFP fluorescence, which served as a readout for compound-induced phenotypic effects. (4) Beads from nanopen in which EGFP fluorescence had disappeared were unloaded and collected into a 96-well plate. (5) The DNA barcode attached to each bead was amplified by PCR and analyzed via NGS to identify the compound. Barcode sequences were mapped to specific compound structures.

### Optimized Protocol for Compound Release from OBOC Beads Using the Beacon System

To facilitate high-resolution phenotypic screening on the Beacon Optofluidic System, we developed and optimized a protocol for compound release from fluorescein-conjugated OBOC beads (Figure 3A). These beads were synthesized using a photolinker strategy and served as a model system to evaluate the efficiency of linker cleavage and compound diffusion within nanoliter-scale nanopen chambers.

**Figure 3:**
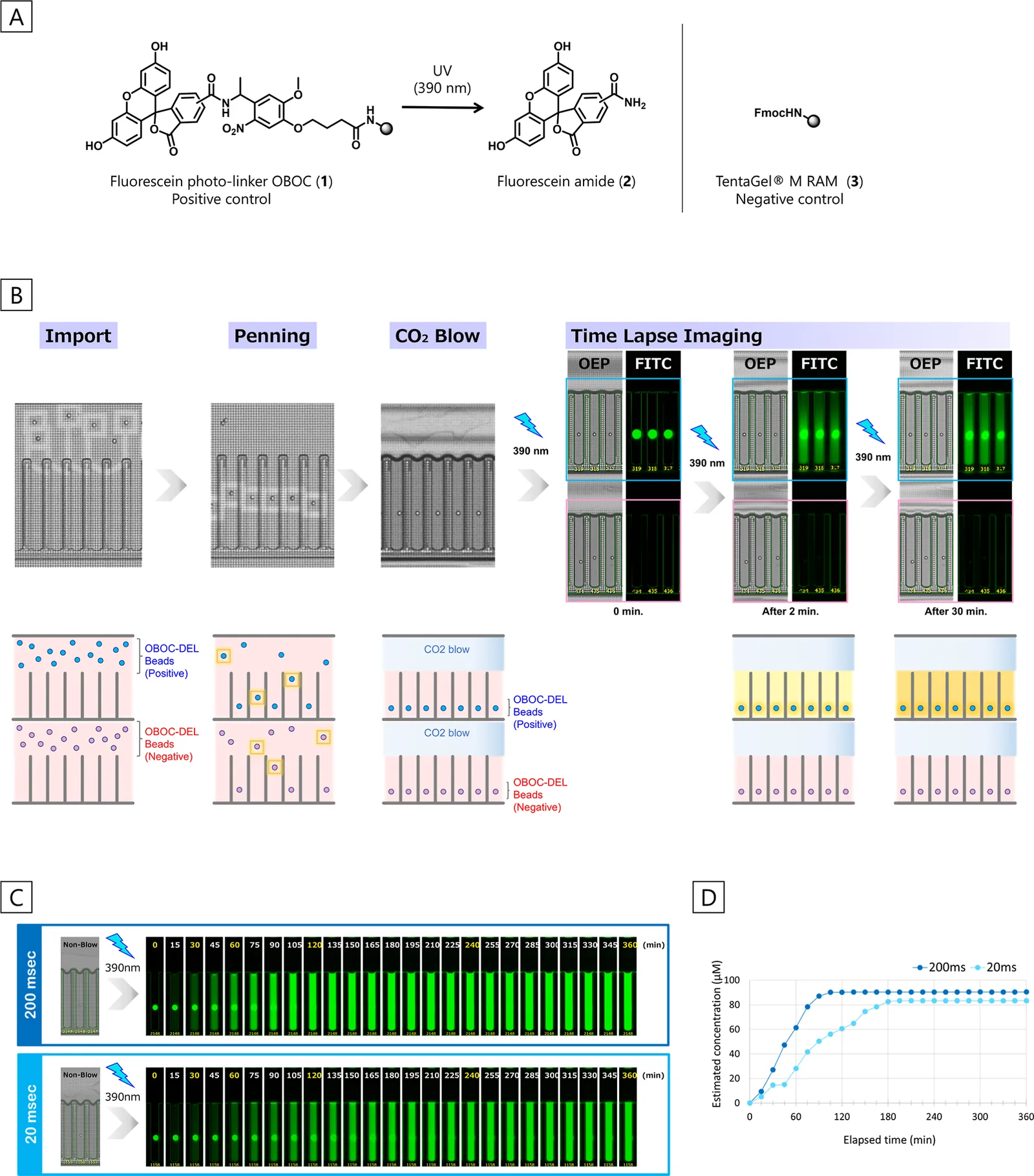
Procedure of the linker cleavage experiment using Beacon with OBOC beads. (A) Schematic of linker cleavage induced by 390 nm light through a DAPI filter. In the validation experiments, the beads were conjugated with a photocleavable linker labeled with fluorescein. (B) OBOC beads adjusted to 1.5 × 10^6^ particles/mL were loaded into the OptoSelect chip and individually penned into nanopens using Opto-ElectroPositioning (OEP). Residual liquid in the channel was removed by manual CO_2_ flow. Linker cleavage was induced by irradiation with 390 nm light through a DAPI filter, applied for 10 s per cycle. FITC fluorescence from released compounds was monitored at defined intervals using time-lapse imaging, with each imaging session preceded by irradiation. In positive beads (top row), FITC fluorescence released from the linker was observed at 2 min. In contrast, negative beads (bottom row) showed no detectable signal under these conditions. (C) Time-lapse images in the FITC channel showed fluorescence release from beads irradiated with either 200 or 20 ms pulses. Longer exposure resulted in a faster increase in fluorescence intensity, indicating accelerated cleavage kinetics. (D) The fluorescein fluorescence intensity at each imaging time point was converted to estimated concentrations using calibration curves. Under 200 ms exposure, fluorescein concentration reached approximately 60 μM at 60 min and exceeded 90 μM at 120 min. With 20 ms exposure, the concentration increased gradually and stabilized at around 80 μM after 180 min.

Fluorescein-conjugated OBOC beads were suspended at a concentration of 1.5 × 10^6^ particles/mL in PBST and loaded into nanopens using optimized parameters for import volume, penning algorithm, and Opto-Electropositioning (OEP). To address bead size variability between synthesis lots, the CNN Cell Detect algorithm was selected, which significantly improved the efficiency of transporting individual particles into nanopen using OEP—a process referred to as penning—as well as counting accuracy. To determine the optimal timing for imaging after compound release, we applied the Wilke–Chang equation to estimate diffusion rates^35^ (Supporting Information 5.2). Assuming fluorescein amide (MW ∼ 400 Da) and a nanopen height of 370 μm, the diffusion time was estimated to be approximately 2 min (Supporting Information 5.3). Accordingly, imaging was initiated ≥ 2 min after UV irradiation, with observation intervals of 30 min to 2 h (Figure 3B).

To quantify released compounds, carboxyfluorescein solutions were used to generate calibration curves correlating fluorescein fluorescence intensity with concentration and exposure time (Figure S9). Theoretical calculations indicated that complete linker cleavage could yield fluorescein concentrations exceeding 100 μM, but such high levels risk cytotoxicity. A concentration of 10 μM was deemed sufficient for phenotypic changes, and imaging conditions were optimized to visualize concentrations up to 50 μM. Exposure times beyond 750 ms caused overexposure, so 500 ms was selected for FITC imaging (Figure S9). Validation experiments using synthesized fluorescein-conjugated (positive) and unconjugated (negative) beads confirmed successful compound release. Positive beads exhibited fluorescence within 1 min, stabilizing around 15 μM after 10 min, while negative beads showed no change. These results demonstrated the feasibility of using OBOC-DEL beads for compound release and subsequent cell-based evaluation (Figure S11). Interestingly, an unexpected pause in the experiment provided us an additional insight that a single 200 ms UV (390 nm) exposure was sufficient to release compounds, with fluorescein fluorescence detected 20 min later. This prompted a reevaluation of exposure duration, showing that shorter exposures could achieve effective cleavage while minimizing phototoxicity.^36^ (Figure S12A).

We also investigated the impact of CO_2_ flow on compound retention. Continuous CO_2_ blowing during assays caused fluorescein leakage into adjacent nanopens, limiting accumulation. In contrast, stopping CO_2_ flow after initial channel clearing resulted in a 5-fold increase in fluorescein concentration—12 μM vs 63 μM after 60 min. This confirmed that halting CO_2_ flow enhances compound retention and concentration within nanopens (Figure S12B, D). Further experiments compared 200 and 20 ms UV (390 nm) exposures. At 200 ms, fluorescein concentrations reached 20 μM in 30 min, 60 μM in 60 min, and exceeded 90 μM in 120 min. At 20 ms, concentrations rose more gradually, reaching 10 μM in 30 min and stabilizing around 80 μM after 180 min. These results demonstrated that UV exposure time could be tuned to control compound release kinetics and concentration (Figure 3C, D).

Based on these findings, we established a standardized workflow for compound release: single synthesized fluorescein-conjugated beads are loaded into nanopens, CO_2_ was applied for >30 min to remove residual liquid and then stopped, UV (390 nm) was irradiated for 200 ms, and FITC imaging was performed at an exposure time of 500 ms for up to 6 h. Fluorescein fluorescence intensity was analyzed using Beacon’s Image Analyzer, and compound concentration was estimated via calibration curves. This optimized protocol ensured efficient and reproducible compound release with minimal cellular damage, forming the foundation for downstream phenotypic screening in the Beacon system.

### Verification of the Effect of Model Compounds on Cellular Phenotype

To validate the Beacon Optofluidic System for phenotypic screening, we conducted a series of experiments to assess both the viability and responsiveness of engineered reporter cells to model compounds. PC-3 cells stably expressing FKBP12^F36V^-EGFP were used as the reporter system.^37^ Although these cells are naturally adherent, they were adapted for microfluidic loading by detachment and handling in nonadherent conditions. Prior to loading, cells were filtered through a 40 μm strainer and suspended at 1.5 × 10^6^ cells/mL. Beacon loading parameters—including cage speed, voltage, and frequency—were optimized to improve penning efficiency, and the CNN Watershed algorithm was applied for accurate cell detection. To establish normal cell behavior, cells were loaded into nanopens and monitored for 16 h without compound exposure. Time-lapse imaging revealed sustained EGFP fluorescence throughout the observation period. Cell proliferation was observed in approximately 13% of nanopens at 6 h, and by 16 h, cell doubling was confirmed in approximately 35% of nanopens. EGFP fluorescence intensity remained stable, with no cells exhibiting values below 5000. Based on these findings, a fluorescence intensity threshold of 5000 was set for subsequent assays to ensure that only viable cells were included in the analysis (Figure 4A, C).

**Figure 4:**
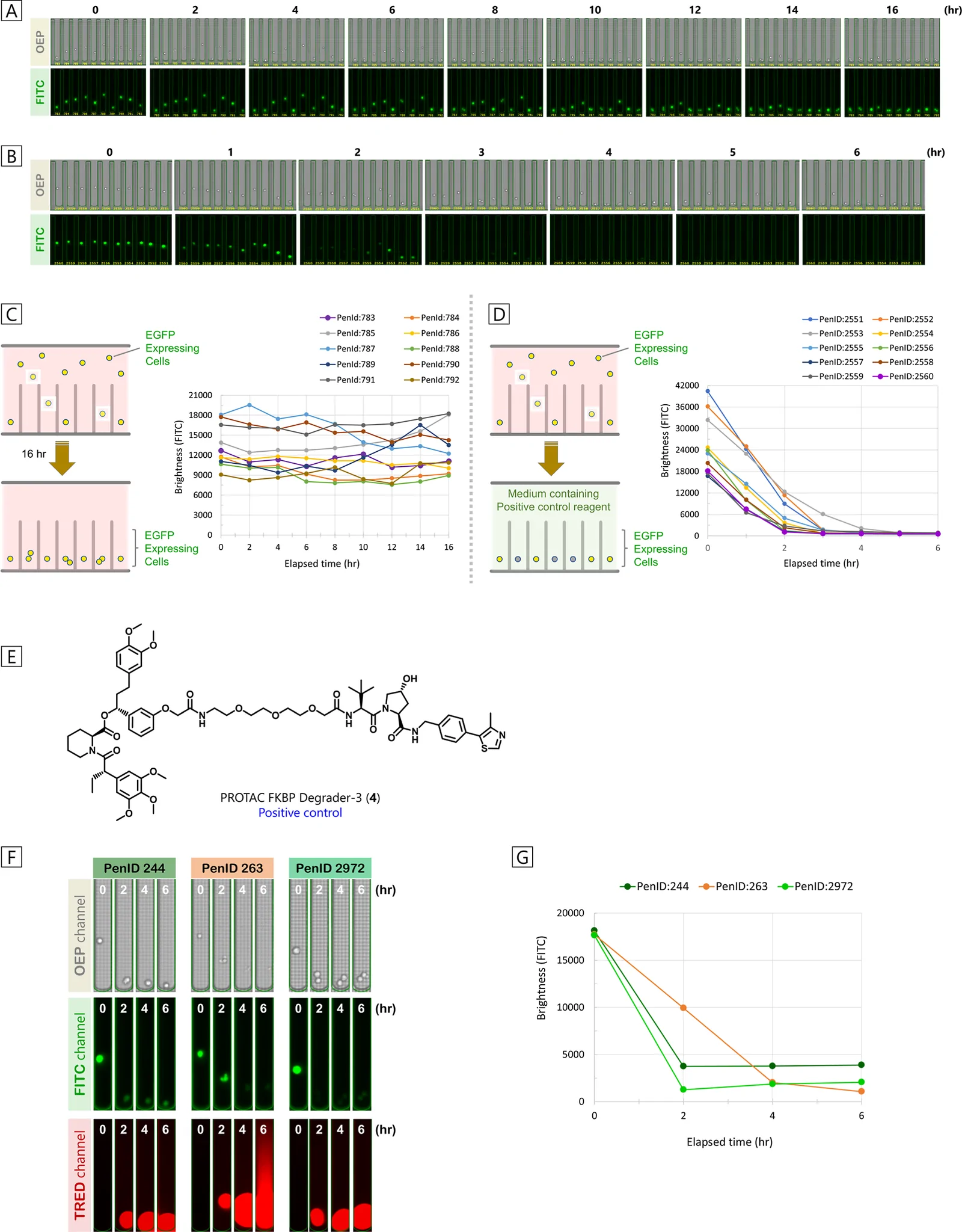
Time-lapse imaging and EGFP fluorescence intensity analysis of single cells on the OptoSelect chip. (A) Single cells were loaded into nanopens on the OptoSelect chip and monitored every 2 h over a 16 h period. Time-lapse imaging revealed sustained EGFP fluorescence in most cells throughout the observation. Cell proliferation was observed in approximately 13% of nanopens at 6 h and in about 35% of nanopens at 16 h. (B) After cell loading, the medium was replaced with one containing the positive control compound (PROTAC FKBP Degrader-3), and EGFP fluorescence intensity was monitored. Time-lapse imaging showed cell proliferation as early as 2 h after the start of observation. (C) Quantitative analysis of EGFP fluorescence intensity in individual cells from (A) indicated that the average intensity remained nearly constant, with no significant decrease observed during the 16 h observation period. (D) Quantitative analysis of individual cells from (B) revealed a marked decrease in EGFP fluorescence, and by 6 h, fluorescence was undetectable in all cells. (E) Chemical structure of PROTAC FKBP Degrader-3. (F) Although nonspecific staining by the viability dye (SYTOX Orange Nucleic Acid Stain) was observed over time, only one nanopen showed widespread fluorescence in the TRED channel after the 6 h assay (e.g., PenID 263). In addition, bright-field imaging in the OEP channel confirmed that live cells retained clear cellular boundaries without morphological distortion (e.g., PenID 244). Combining these features from both channels allowed clear discrimination between live and dead cells, and overall viability exceeded 99%. Furthermore, cell proliferation was observed in approximately 12% of nanopens (e.g., PenID 2972). (G) During the 6 h observation period, differences in cellular behavior were observed at the single-cell level, including cells showing either rapid or gradual decreases in EGFP fluorescence intensity.

Next, to verify the system’s ability to detect compound-induced protein degradation, we introduced a model compound—PROTAC FKBP Degrader-3 ^37,38^ —at a final concentration of 1.0 μM (Figure 4E). Cells were cultured for 6 h, and EGFP fluorescence intensity was monitored every 2 h. Given the relatively large molecular weight of PROTAC FKBP Degrader-3 (MW = 1295.54), diffusion within the nanopen is expected to be slower than that of small-molecule probes, with an estimated diffusion time of approximately 5 min (Supporting Information 5.3). However, this modest delay does not affect the experimental workflow, as phenotypic changes were evaluated over much longer time scales (hours). A marked decrease in EGFaP fluorescence was observed as early as 2 h, and by 6 h, fluorescence was nearly undetectable across all cells. Despite initial variability in EGFP levels, the decline pattern was consistent, confirming robust compound-induced degradation (Figure 4B, D). The potency of FKBP Degrader-3 was evaluated independently, and the DC_50_ was determined to be 44 nM (Supporting Information 3.2). At the concentrations applied in this study, FKBP Degrader-3 induced robust and reproducible degradation of the FKBP12–EGFP reporter, which could be reliably detected at the single-cell level using the PhenoDEL workflow. Notably, because this proof-of-concept assay relies on an EGFP-tagged reporter, loss of fluorescence reflects attenuation of the reporter signal and does not necessarily indicate direct degradation of the endogenous target protein. Previous studies have demonstrated that EGFP contains ubiquitinatable lysine residues (e.g., Lys158), and that ubiquitination and turnover of protein tags themselves can contribute to apparent degradation events, potentially leading to false-positive readouts in degradation-based assays.^39^ Accordingly, particularly in screening campaigns involving unknown compounds, EGFP signal loss should be interpreted with caution as a reporter-based phenotypic readout rather than as definitive evidence of endogenous target protein degradation.

Cell viability in this study was assessed using SYTOX Orange Nucleic Acid Stain, a membrane-impermeable dye that selectively labels cells with compromised plasma membrane integrity. The Beacon Optofluidic System currently supports fluorescence-based imaging but does not accommodate luminescence detection; therefore, ATP-based luminescent assays such as CellTiter-Glo could not be implemented concurrently within the same nanopen-based workflow. While CellTiter-Glo is well suited for detecting early metabolic perturbations and cytostatic effects, SYTOX Orange is specifically sensitive to overt cytotoxicity associated with loss of membrane integrity. Accordingly, the present study focuses on excluding compounds that induce acute cell death rather than detecting nonlethal growth inhibition or early metabolic stress. Under these conditions, changes in EGFP fluorescence can be interpreted independently of gross cytotoxicity. Having confirmed degradation, we next assessed cytotoxicity by adding SYTOX Orange Nucleic Acid Stain (final concentration, 2.0 μM) after 22 h of incubation. Weak fluorescence was detected in the TRED channel; however, bright-field imaging revealed that most cells retained well-defined boundaries and exhibited no morphological alterations, suggesting that staining was largely nonspecific and membrane integrity remained intact. In contrast, cell death was observed in 2–3 cells per field (1.4–2.0%), characterized by loss of boundaries and diffuse TRED fluorescence. Notably, approximately 43% of nanopens displayed cell proliferation at 22 h, and combined with a viability exceeding 98%, these findings demonstrate that the compound promotes protein degradation with minimal impact on cell viability (Figure S16).

Based on these findings, the assay conditions were optimized for small-molecule screening. A combined medium containing PROTAC FKBP Degrader-3 and SYTOX Orange was prepared, and cells were cultured for 6 h following a single UV irradiation (390 nm, 200 ms). Imaging was performed every 2 h using exposure times of 2000 ms for the FITC channel and 1000 ms for the TRED channel. During the assay, cell proliferation was observed in 11–14% of nanopens. At the 6 h time point, strong SYTOX Orange fluorescence was detected; however, except for a single case, the signal did not spread throughout the nanopen. Bright-field imaging confirmed that most cells maintained clear morphology without distortion. These observations indicate that, despite some nonspecific staining, reliable discrimination between live and dead cells was achieved, and overall cell viability exceeded 99% after the assay. This approach enabled single-cell analysis of diverse behaviors, including subtle differences in cell size and protein expression intensity prior to the assay, presence or absence of cell division during the assay, sedimentation patterns within nanopens, and variability in EGFP fluorescence decay rates (Figure 4F, G). In light of these preliminary results, the concentration of the viability dye was reduced in subsequent experiments to minimize nonspecific staining of live cells and prevent signal saturation in the TRED channel.

Collectively, these results indicate that the Beacon system enables high-resolution phenotypic evaluation of compound activity while minimizing phototoxicity. The engineered PC-3 cells demonstrated stable behavior on the Beacon chip and responded predictably to the model compound. The optimized assay conditions—including cell loading, compound exposure, and imaging protocols—established a reliable platform for evaluating cellular phenotypes in response to small molecules, supporting the use of Beacon for single-cell screening and laying the groundwork for broader application in drug discovery. As described above, because this assay relies primarily on fluorescence changes of an EGFP-tagged reporter as the primary readout, validation using orthogonal assays that directly assess endogenous target protein levels is essential for accurate interpretation of screening results. Based on these data, we note that PhenoDEL is currently best suited for the detection of compounds with relatively strong degradation activity, while further optimization and evaluation may be required to reliably resolve weaker effects at the single-cell level under the present conditions.

### Validation of the Single-Bead, Single-Cell Coculture Screening Workflow

To establish a high-resolution phenotypic screening platform using the Beacon Optofluidic System, we developed a workflow that enables precise 1:1 coculture of single OBOC-DEL beads and individual reporter cells within nanoliter-scale nanopens. This approach, central to the PhenoDEL concept, allows direct observation of compound-induced cellular responses and identification of active compounds via DNA barcoding and sequencing. In this study, PROTAC FKBP Degrader-3 photolinker OBOC-DEL beads (**5**) and compound unconjugated OBOC-DEL beads (**6**) were synthesized via solid-phase synthesis and DNA conjugation, incorporating a photocleavable linker for compound release upon UV (390 nm) irradiation. Each bead carried a unique compound and DNA barcode, allowing for precise tracking and identification following phenotypic screening (Figure 5A).

**Figure 5:**
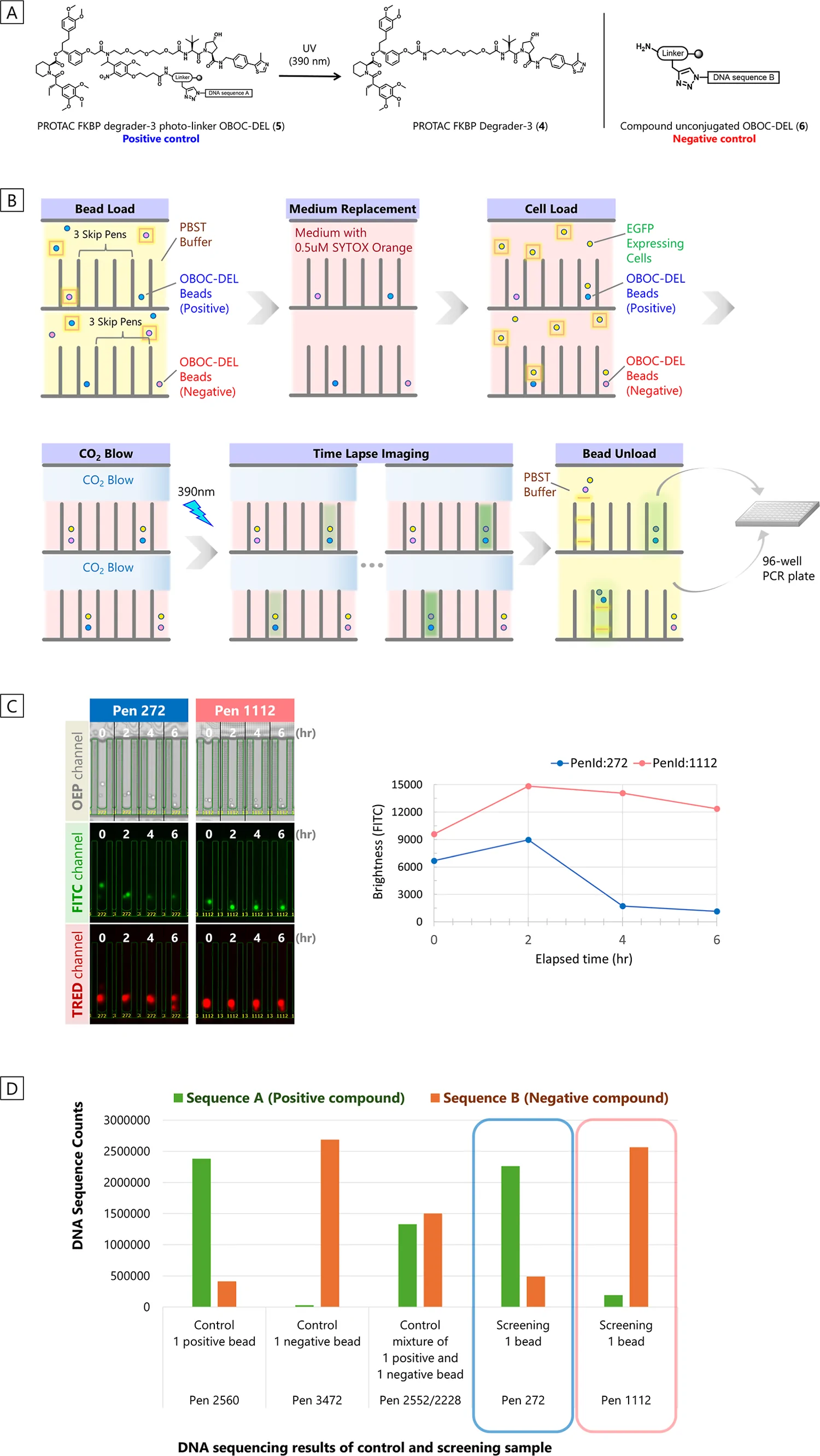
Evaluation of cell based screening using on-bead compounds on the Beacon platform. (A) Chemical structure of the PROTAC FKBP Degrader-3 photolinker OBOC-DEL beads and compound unconjugated OBOC-DEL beads. The photocleavable linker enables compound release upon UV irradiation. Each bead carries a unique compound and a corresponding DNA barcode, allowing for postscreening identification of hit compounds via sequencing. (B) Experimental workflow on the Beacon system. A single cell was loaded into a nanopen containing one OBOC-DEL bead. To prevent compound diffusion outside the nanopen, the medium in the channel was removed. After UV (390 nm) irradiation, cells were monitored for 6 h. Nanopens containing cells with or without changes in EGFP fluorescence intensity were selected, and beads from these pens were recovered for NGS analysis. (C) Phenotypic screening of 1:1 OBOC-DEL bead–cell cocultures using Beacon. Nanopens containing one bead and one cell were imaged every 2 h after UV (390 nm) irradiation. Most cells exhibited clear boundaries (OEP) and were not stained by the nucleic acid staining reagent (TRED), indicating stable survival within the nanopen. In some cells, EGFP fluorescence intensity decreased, while others showed little to no change even after 6 h. After 6 h of culture, PenID 272 showed a decrease in brightness, whereas PenID 1112 exhibited no notable change. (D) Graphs showing DNA sequencing results. From left to right: positive control (PenID 2560), negative control (PenID 3472), a control containing a 1:1 mixture of positive and negative beads (PenID 2552/2228), a screened single bead (PenID 272), and a screened single bead (PenID 1112). NGS analysis showed that single beads recovered from a nanopen (PenID 272) exhibiting time-dependent cellular fluorescence changes predominantly identified sequence A (positive compound), whereas those from a nanopen (PenID 1112) with no observable fluorescence change predominantly identified sequence B (negative compound).

Within the Beacon workflow, individual OBOC-DEL beads were loaded into nanopens, followed by single FKBP12^F36V^-EGFP reporter cells. After confirming successful penning of both bead and cell, UV (390 nm) irradiation (200 ms) was applied to cleave the compound from the bead. The released compound diffused into the nanopen and interacted with the cocultured cell. Time-lapse imaging was performed every 2 h over a 6 h period to monitor changes in EGFP fluorescence, serving as a readout for compound activity. Images acquired during the assay were analyzed using Beacon’s image analysis software to extract data on cellular morphology and EGFP fluorescence intensity. Beads from nanopens showing significant phenotypic changes—defined as a EGFP fluorescence intensity decrease of more than 5000 units—were recovered and transferred to a 96-well plate. The DNA barcode region of each bead was amplified by PCR and subjected to NGS to identify the compound responsible for the observed effect (Figure 5B).

To ensure objective classification, only nanopens containing one bead and one viable cell with clearly detectable EGFP expression before the assay were included in the analysis. Viable cells were defined as those showing distinct boundaries without deformation in the OEP channel and lacking fluorescence diffusion in the TRED channel. This approach confirmed that changes in EGFP fluorescence were caused by compound activity, not by cell damage (Figure S19).

During the screening, most cells remained viable and showed no signs of damage. In FITC channel imaging, some cells exhibited a marked decrease in EGFP fluorescence, while others showed minimal change. This variability may suggest differential activity of compounds encoded on the beads. In the TRED channel, fluorescence signals were observed on the OBOC-DEL beads due to SYTOX staining of DNA barcodes present on the bead surface. However, these signals were distinguishable from cellular fluorescence based on differences in size and circularity, allowing clear discrimination from cells. To correlate phenotypic outcomes with compound identity, beads were recovered from nanopens representing both positive and negative responses and analyzed by NGS (Figure 5C). To validate the specificity of the assay, control experiments were performed in which OBOC-DEL beads were loaded and unloaded without coculturing with cells. DNA barcodes from these beads were sequenced to establish baseline profiles. The analyzed beads included those with compounds covalently attached via photocleavable linkers (positive beads), beads without any compound conjugation (negative beads), and a mixed population containing both types at a 1:1 ratio. Recovered beads were subjected to two rounds of PCR amplification, and the resulting products were sequenced using Illumina high-throughput sequencing. The data were processed and analyzed to determine enrichment patterns and identify active compounds. Comparative analysis of sequencing data from control and test samples confirmed the specificity and reproducibility of the screening workflow (Figure 5D).

This integrated approach—combining compound release, phenotypic tracking, and molecular decoding—demonstrates the power of Beacon-based PhenoDEL screening. It enables high-resolution, activity-based screening of DNA-encoded libraries at the single-cell level, overcoming limitations of conventional droplet-based methods such as lack of spatial control, inability to track individual cell behavior, and difficulty in eliminating false positives. By enabling precise 1:1 pairing of beads and cells, real-time tracking of cellular responses, and direct linkage between phenotype and compound identity, this workflow supports scalable and mechanism-driven drug discovery. It is compatible with various cell types and reporter systems, making it a versatile platform for functional genomics and small molecule screening.

## Conclusions

In this study, we developed and named a novel screening strategy, PhenoDEL (Phenotypic DNA-Encoded Library), which integrates OBOC-DEL with the Beacon optofluidic system to enable phenotype-driven identification of small molecules that induce target protein degradation. By coculturing individual beads and cells within nanoliter-scale nanopen chambers, this platform enables high-resolution, activity-based screening at the single-cell level.

A defining feature of PhenoDEL is its deliberate emphasis on single-cell resolution, temporal phenotyping, and causal interpretability. Compared with traditional HTS and conventional DEL approaches,^14,40–42^ the current implementation achieves more limited library diversity and throughput; however, it provides a unique capability for single-cell-resolved phenotypic screening. PhenoDEL therefore occupies a conceptually distinct position, enabling time-resolved single-cell analysis and direct linkage between compound identity and intracellular phenotypic response—capabilities that are not accessible through population-averaged screening methods. In light of these considerations, PhenoDEL is particularly amenable to focused libraries designed to interrogate specific targets, pathways, or phenotypic hypotheses, rather than large, maximally diverse screening collections.

Using an FKBP12^F36V^-based degradation model as a proof of concept, we demonstrated robust single-cell phenotypic screening and successful identification of degradation-inducing compounds. The Beacon system enables precise 1:1 loading of beads and cells with visual confirmation by real-time imaging, allowing exclusion of dead or damaged cells and improving the reliability of phenotypic readouts. Notably, compound release from OBOC-DEL beads could be finely controlled by brief UV irradiation, and assay sensitivity was further enhanced by optimizing on-chip compound retention.

PhenoDEL is compatible with a range of cell types, including suspension cells (e.g., CHO-S), adherent cells (e.g., HEK-293), and hybridoma cells (e.g., OKT3). Consistent with this, Beacon-based optofluidic platforms have been reported to support diverse cell types and assay modalities across multiple biological applications.^32,43–45^ For scalable screening, the Beacon system supports parallel operation of up to four OptoSelect 20k chips, each containing approximately 20,000 nanopens, enabling screening of up to ∼80,000 individual bead–cell pairs in a single run.

Although further optimization and incorporation of orthogonal validation assays will be required—particularly given the use of fluorescent protein–tagged reporters in the current implementation—PhenoDEL establishes a general framework for future activity-based discovery efforts in which throughput is consciously balanced against information density and mechanistic insight. By enabling high-resolution, single-cell phenotypic screening, PhenoDEL provides a promising foundation for next-generation drug discovery and functional genomics, including the identification of protein degraders and other mechanism-driven small molecules.

## Supplementary Material



## Notes

Detailed experimental procedures; materials and methods; compound synthesis protocols; cell preparation; studies on compound release from OBOC-DEL beads in the Beacon system; validation of compound-induced cellular phenotypes; coculture assays of OBOC-DEL beads and cells; and all supplementary figures and tables (PDF)

## ∥

Novartis Pharma K.K., Tokyo 105-6333, Japan

## ⊥

Reborna Biosciences, Inc., Kanagawa 251-8555, Japan

## #

Mediford Corporation, Tokyo 174-0053, Japan

## ¶

PeptiDream Inc., Kanagawa 210-0821, Japan

## ▽

Philochem AG, Libernstrasse 3, Otelfingen CH-8112, Switzerland

## References

[1] Chamberlain P. P., Hamann L. G. (2019). Development of targeted protein degradation therapeutics. Nat. Chem. Biol..

[2] Tsai J. M., Nowak R. P., Ebert B. L., Fischer E. S. (2024). Targeted protein
degradation: from mechanisms to clinic. Nat.
Rev. Mol. Cell Biol..

[3] Zhong G., Chang X., Xie W., Zhou X. (2024). Targeted protein degradation:
advances in drug discovery and clinical practice. Signal Transduction Targeted Ther..

[4] Liu X., Ciulli A. (2023). Proximity-Based Modalities
for Biology and Medicine. ACS Cent. Sci..

[5] Singh S., Tian W., Severance Z. C., Chaudhary S. K., Anokhina V., Mondal B., Pergu R., Singh P., Dhawa U., Singha S., Choudhary A. (2023). Proximity-inducing
modalities: the past, present, and future. Chem.
Soc. Rev..

[6] Dong G., Ding Y., He S., Sheng C. (2021). Molecular Glues for
Targeted Protein Degradation: From Serendipity to Rational Discovery. J. Med. Chem..

[7] Holdgate G. A., Bardelle C., Berry S. K., Lanne A., Cuomo M. E. (2024). Screening
for molecular glues – Challenges and opportunities. SLAS Discovery.

[8] Domostegui A., Nieto-Barrado L., Perez-Lopez C., Mayor-Ruiz C. (2022). Chasing molecular
glue degraders: screening approaches. Chem.
Soc. Rev..

[9] Darling S., Bajrami I., West S. C. (2025). From serendipity
to strategy: molecular
glue degraders in cancer therapeutics. Crit.
Rev. Biochem. Mol. Biol..

[10] Neri D., Lerner R. A. (2018). DNA-Encoded Chemical
Libraries: A Selection System
Based on Endowing Organic Compounds with Amplifiable Information. Annu. Rev. Biochem..

[11] Favalli N., Bassi G., Scheuermann J., Neri D. (2018). DNA-encoded chemical
libraries – achievements and remaining challenges. FEBS Lett..

[12] Goodnow R. A., Dumelin C. E., Keefe A. D. (2017). DNA-encoded chemistry:
enabling the deeper sampling of chemical space. Nat. Rev. Drug Discovery.

[13] Gironda-Martínez A., Donckele E. J., Samain F., Neri D. (2021). DNA-Encoded Chemical
Libraries: A Comprehensive Review with Successful Stories and Future
Challenges. ACS Pharmacol. Transl. Sci..

[14] Valeur E., Jimonet P. (2018). New Modalities, Technologies, and
Partnerships in Probe
and Lead Generation: Enabling a Mode-of-Action Centric Paradigm. J. Med. Chem..

[15] Disch J. S., Duffy J. M., Lee E. C. Y., Gikunju D., Chan B., Levin B., Monteiro M. I., Talcott S. A., Lau A. C., Zhou F., Kozhushnyan A., Westlund N. E., Mullins P. B., Yu Y., von Rechenberg M., Zhang J., Arnautova Y. A., Liu Y., Zhang Y., McRiner A. J., Keefe A. D., Kohlmann A., Clark M. A., Cuozzo J. W., Huguet C., Arora S. (2021). Bispecific
Estrogen Receptor α Degraders Incorporating Novel Binders Identified
Using DNA-Encoded Chemical Library Screening. J. Med. Chem..

[16] Chen Q., Liu C., Wang W., Meng X., Cheng X., Li X., Cai L., Luo L., He X., Qu H., Luo J., Wei H., Gao S., Liu G., Wan J., Israel D. I., Li J., Dou D. (2023). Optimization of PROTAC Ternary Complex Using DNA Encoded
Library Approach. ACS Chem. Biol..

[17] Mason J. W., Chow Y. T., Hudson L., Tutter A., Michaud G., Westphal M. V., Shu W., Ma X., Tan Z. Y., Coley C. W., Clemons P. A., Bonazzi S., Berst F., Briner K., Liu S., Zécri F. J., Schreiber S. L. (2024). DNA-encoded library-enabled discovery of proximity-inducing
small molecules. Nat. Chem. Biol..

[18] Liu S., Tong B., Mason J. W., Ostrem J. M., Tutter A., Hua B. K., Tang S. A., Bonazzi S., Briner K., Berst F., Zécri F. J., Schreiber S. L. (2023). Rational
Screening for Cooperativity in Small-Molecule Inducers of Protein–Protein
Associations. J. Am. Chem. Soc..

[19] Petersen L. K., Christensen A. B., Andersen J., Folkesson C. G., Kristensen O., Andersen C., Alzu A., Sløk F. A., Blakskjær P., Madsen D., Azevedo C., Micco I., Hansen N. J. V. (2021). Screening of DNA-Encoded Small Molecule Libraries inside
a Living Cell. J. Am. Chem. Soc..

[20] MacConnell A. B., McEnaney P. J., Cavett V. J., Paegel B. M. (2015). DNA-encoded
solid-phase
synthesis: encoding language design and complex oligomer library synthesis. ACS Comb. Sci..

[21] Mendes K. R., Malone M. L., Ndungu J. M., Suponitsky-Kroyter I., Cavett V. J., McEnaney P. J., MacConnell A. B., Doran T. M., Ronacher K., Stanley K., Utset O., Walzl G., Paegel B. M., Kodadek T. (2017). High-throughput identification
of DNA-encoded IgG ligands that distinguish active and latent Mycobacterium
tuberculosis infections. ACS Chem. Biol..

[22] Cochrane W. G., Malone M. L., Dang V. Q., Cavett V., Satz A. L., Paegel B. M. (2019). Activity-based DNA-encoded
library screening. ACS Comb. Sci..

[23] Dixit A., Paegel B. M. (2026). Solid-phase DNA-encoded library synthesis:
a master
builder’s instructions. Nat. Protoc..

[24] Barhoosh H., Dixit A., Cochrane W. G., Cavett V., Prince R. N., Blair B. O., Ward F. R., McClure K. F., Patten P. A., Paulick M. G., Paegel B. M. (2024). Activity-Based
DNA-Encoded Library
Screening for Selective Inhibitors of Eukaryotic Translation. ACS Cent. Sci..

[25] Bushman J. W., Deng W., Samarasinghe K. T. G., Liu H.-Y., Li S., Vaish A., Golkar A., Ou S.-C., Ahn J., Harijan R. K., Han H., Duffy T., Imler E., Wu J., Rawson R., Liu L., Ndungu J. M., Hocker M. D., Roy A., Senese A. D., Lin C.-C., Tran D., Campos A., Blanco G., Toth J. I., Velentza A., Burritt A., Parker G. S., Thompson P. A., Bailey S., den Besten W., Voss S., Zhou B., Ashton K. S., Bedel O., Min J., Potts P. R. (2025). Discovery of a VHL molecular glue degrader of GEMIN3
by Picowell RNA-seq. bioRxiv.

[26] Paegel, B. M. ; MacConnell, A. B. ; Kodadek, T. ; McEnaney, P. J. Encoded Solid Phase Compound Library with Polynucleotide Based Barcoding. WO 2018/089641, 2018.

[27] Rokicki, J. F. ; Nguyen, M. V. ; Vijayan, K. ; MacConnell, A. B. Oligonucleotide Encoded Chemical Libraries. WO 2019/060830, 2019.

[28] Cayer, D. ; MacConnell, A. ; Chubukov, P. ; Ramji, R. ; Stromberg, S. Methods and Systems for Microfluidic Screening. WO 2021/072306, 2021.

[29] Abeywardane, A. A. ; Apsunde, T. D. ; Etienne, G. N. ; Gambardella, A. ; Mainquist, J. K. ; Meyer, A. J. ; Minie, B. ; Paula, S. ; Vickers, D. A. L. ; Yamada, K. High Throughput Screening in Droplets. WO 2022/190014, 2022.

[30] Mocciaro A., Roth T. L., Bennett H. M., Soumillon M., Shah A., Hiatt J., Chapman K., Marson A., Lavieu G. (2018). Light-activated cell identification and sorting (LACIS)
for selection of edited clones on a nanofluidic device. Commun. Biol..

[31] Jorgolli M., Nevill T., Winters A., Chen I., Chong S., Lin F.-F., Mock M., Chen C., Le K., Tan C., Jess P., Xu H., Hamburger A., Stevens J., Munro T., Wu M., Tagari P., Miranda L. P. (2019). Nanoscale integration of single cell
biologics discovery
processes using optofluidic manipulation and monitoring. Biotechnol. Bioeng..

[32] Le K., Tan C., Le H., Tat J., Zasadzinska E., Diep J., Zastrow R., Chen C., Stevens J. (2020). Assuring clonality
on the Beacon Digital Cell Line Development Platform. Biotechnol. J..

[33] Winters A., McFadden K., Bergen J., Landas J., Berry K. A., Gonzalez A., Salimi-Moosavi H., Murawsky C. M., Tagari P., King C. T. (2019). Rapid single B cell
antibody discovery using nanopens
and structured light. mAbs.

[34] Xiong T., Wang G., Yu P., Li Z., Li D., Zhang J., Lu S., Yang R., Lian X., Mi J., Ma R., Li Z., Marcucci G., Zhao T., Caligiuri M. A., Yu J. (2025). CAR-T cells targeting CD155 reduce
tumor burden in preclinical models of leukemia and solid tumors. J. Clin. Invest..

[35] Wilke, C. R. ; Chang, P. Correlation of diffusion coefficients in dilute solutions; Wiley, 1955.

[36] Icha J., Weber M., Waters J. C., Norden C. (2017). Phototoxicity in live
fluorescence microscopy, and how to avoid it. BioEssays.

[37] Ottis P., Toure M., Cromm P. M., Ko E., Gustafson J. L., Crews C. M. (2017). Assessing different E3 ligases for
small molecule induced
protein ubiquitination and degradation. ACS
Chem. Biol..

[38] Bradner, J. ; Roberts, J. ; Nabet, B. ; Winter, G. ; Phillips, A. J. ; Heffernan, T. P. ; Buckley, D. Targeted protein degradation to attenuate adoptive T-cell therapy associated adverse inflammatory responses. WO 2017/024318, 2017.

[39] Zeng M., Xiong Y., Safaee N., Nowak R. P., Donovan K. A., Yuan C. J., Nabet B., Gero T. W., Feru F., Li L., Gondi S., Ombelets L. J., Quan C., Jänne P. A., Kostic M., Scott D. A., Westover K. D., Fischer E. S., Gray N. S. (2020). Exploring targeted degradation strategy for oncogenic
KRAS G12C. Cell Chem. Biol..

[40] Macarron R., Banks M. N., Bojanic D., Burns D. J., Cirovic D. A., Garyantes T., Green D. V. S., Hertzberg R. P., Janzen W. P., Paslay J. W., Schopfer U., Sittampalam G. S. (2011). Impact
of high-throughput screening in biomedical research. Nat. Rev. Drug Discovery.

[41] Franzini R. M., Neri D., Scheuermann J. (2014). DNA-Encoded Chemical Libraries: Advancing
beyond Conventional Small-Molecule Libraries. Acc. Chem. Res..

[42] Dragovich P. S., Haap W., Mulvihill M. M., Plancher J.-M., Stepan A. F. (2022). Small-Molecule
Lead-Finding Trends across the Roche and Genentech Research Organizations. J. Med. Chem..

[43] Zhang Y., Park M., Ghoda L. Y., Zhao D., Valerio M., Nafie E., Gonzalez A., Ly K., Parcutela B., Choi H., Gong X., Chen F., Harada K., Chen Z., Nguyen L. X. T., Pichiorri F., Chen J., Song J., Forman S. J., Amanam I., Zhang B., Jin J., Williams J. C., Marcucci G. (2024). IL1RAP-specific
T cell engager depletes acute myeloid leukemia stem cells. J. Hematol. Oncol..

[44] Colina A. S., Shah V., Shah R. K., Kozlik T., Dash R. K., Terhune S., Zamora A. E. (2024). Current
advances in experimental
and computational approaches to enhance CAR T cell manufacturing protocols
and improve clinical efficacy. Front. Mol. Med..

[45] Rienzo M., Lin K.-C., Mobilia K. C., Sackmann E. K., Kurz V., Navidi A. H., King J., Onorato R. M., Chao L. K., Wu T., Jiang H., Valley J. K., Lionberger T. A., Leavell M. D. (2021). High-throughput optofluidic screening
for improved
microbial cell factories via real-time micron-scale productivity monitoring. Lab Chip.

